# All-trans retinoic acid attenuates the progression of Ang II-induced abdominal aortic aneurysms in ApoE^−/−^mice

**DOI:** 10.1186/s13019-020-01208-w

**Published:** 2020-07-02

**Authors:** Jie Xiao, Jinping Liu, Iohang Lio, Chuanlei Yang, Xing Chen, Hua Zhang, Shuxia Wang, Zhanjie Wei

**Affiliations:** 1 Department of Cardiovascular Surgery, Central Hospital of Wuhan, Huazhong University of Science and Technology, Wuhan, 430014 China; 2 Department of Cardiovascular Surgery, Zhongnan Hospital, Wuhan University, Wuhan, 430071 Hubei China; 3 Department of Cardiovascular Surgery, Union Hospital, Tongji Medical College, Huazhong University of Science and Technology, Wuhan, 430022 China; 4 Department of Radiology, Central Hospital of Wuhan, Huazhong University of Science and Technology, Wuhan, 430014 China; 5 Department of General Surgery, Central Hospital of Wuhan, Huazhong University of Science and Technology, Wuhan, 430014 China

**Keywords:** Abdominal aortic aneurysm, All-trans retinoic acid (ATRA), Inflammation

## Abstract

**Background:**

To determine whether all-trans retinoic acid (ATRA) can influence the development of Angiotensin II (Ang II) induced experimental abdominal aortic aneurysms (AAAs).

**Methods:**

Apolipoprotein E knock-out (ApoE^−/−^) mice were randomly assigned to 4 groups. Mice in the AAA and ATRA groups underwent continuous subcutaneous Ang II infusion for 28 days to induce AAA, while the Sham and Control groups were infused with saline. Systolic blood pressure was measured by the tail-cuff technique. The Control and ATRA groups received ATRA treatment. Aortic tissue samples were obtained at 28 days after surgery and evaluated by aortic diameter measurement, Western blotting, immunohistochemistry, and hematoxylin-eosin (H&E) and Verhoeff-Van Gieson (EVG) staining.

**Results:**

The abdominal aortic diameter was significantly reduced in the ATRA group compared with the AAA group (3 of 12 (25%) vs 9 of 12 (75%), *P* < 0.05), and the ATRA group exhibited reduced blood pressure on days 7, 14, and 28. Low expression of angiotensin II receptor type 1 (AT1), matrix metalloproteinase 2 (MMP2), and matrix metalloproteinase 9 (MMP9) and EVG staining revealed a significant reduction in the disruption of elastic fibers in the abdominal aortic tissue of the ATRA group compared to the AAA group. Western blot analysis indicated that protein levels of retinoic acid receptor α (RARα), MMP2, MMP9, and AT1 were dramatically affected by ATRA treatment.

**Conclusions:**

In conclusion, ATRA attenuates the progression of Ang II-induced AAAs, possibly by downregulating MMP2, MMP9, and AT-1 expression.

## Background

The global mortality due to abdominal aortic aneurysms (AAAs) is high, and AAAs are believed to be associated with age-related degenerative disease. Several risk factors significantly correlate with AAA, including advanced age, male gender, smoking, high lipid levels, hypertension, obesity, and hyperhomocysteinemia (HHcy) [1, 2]. For adults over the age of 60 years, the prevalence of AAA is approximately 5%. The detection rate of AAA has increased due to more efficient observation and screening methods. When AAA patients have a vessel diameter greater than 5.5 cm, the increased risk of rupture can require elective surgery or endovascular repair. If an acute ruptured AAA does not receive timely treatment, the mortality rate can reach 100%. Routine abdominal screening detects large numbers of asymptomatic AAAs. In 90% of patients, these AAAs have diameters smaller than 5.5 cm, and although they can gradually expand, the risk of rupture is relatively low; additionally, there are no clear strategies to treat these AAAs [3].

Histopathologically, AAA is characterized by reduced elastic fibers, significant elastorrhexis, and the destruction and disruption of the integrity of the extracellular matrix accompanied by apoptosis of medial vascular smooth muscle cells (VSMCs), infiltration of macrophages and lymphocytes, and neovascularization [4]. Inflammation and matrix degradation in the aneurysm microenvironment have been implicated in aneurysm wall weakening and are crucial for AAA formation and rupture. Matrix metalloproteinase (MMP) expression and VSMC apoptosis are regulated by inflammatory responses in the aneurysm microenvironment [5, 6]. Diverse pathogenicity factors have been described in AAA, including smoking, high blood pressure, and obesity. These risk factors are similar to those for atherosclerosis, and the intimal pathology of atherosclerosis and thrombosis are also similar to that of AAA [7].

All-trans retinoic acid (ATRA) is the active metabolite of vitamin A and is a lipophilic small molecule (300 Da) that binds albumin and has a blood concentration of 1–10 nmol/L [8]. ATRA is primarily used to treat promyelocytic leukemia [9], as well as dermatosis and certain types of solid tumors [10]. ATRA binds to retinoic acid receptors (RARs) and retinoid X receptors (RXRs) with high affinity. RARs/RXRs are widely distributed in cardiovascular tissue [11], and RARα has been shown to reduce aortic atherosclerosis in Apolipoprotein E knockout (ApoE^−/−^) mice. ATRA inhibits the protein expression and activity of the metalloproteinases matrix metalloproteinase 2 and 9 (MMP2 and MMP9) while the controlling proliferation and migration of arterial smooth muscle cells and endothelial cells [12]. Previous studies have demonstrated that plasma MMP-2 and MMP-9 levels are elevated in AAAs [13]. Thus, inhibiting the expression of MMP-2 and MMP-9 may affect the formation of AAA. ATRA can inhibit the inflammatory response by regulating the proliferation of T cells [14], B cells [15], and macrophages, as well as the secretion of C-reactive protein (CRP). ATRA can block angiogenesis through the retinoid-mediated reduction in the expression of vascular endothelial growth factor, which is a potent angiogenic factor. While many studies have examined the use of ATRA to treat atherosclerosis [16], no studies have examined the treatment of AAAs with ATRA.

No effective treatment has been identified for AAA beyond the early stages, and a non-invasive treatment is of great interest. The properties of ATRA described above suggest that it may be beneficial for the treatment of AAA. We hypothesized that treatment with ATRA attenuates the progression of Angiotensin II (Ang II)-induced AAA by inhibiting the expression of MMP2, MMP9, and angiotensin II receptor type 1 (AT1) in ApoE^−/−^ mice.

## Methods

### Animal model

A total of 40 male ApoE^−/−^ adult mice were obtained from the Beijing HFK Bioscience Co. Ltd. (HFK). Mice aged 16 weeks and with a body weight of 28–30 g were used in the experiments. All mice were housed under environmentally controlled specific pathogen-free conditions with a 12:12-h light-dark cycle. The mice were fed a standard laboratory chow and tap water ad libitum. All surgical procedures were performed with general anesthesia based on previously described methods [17]. Osmotic pumps (Alzet Osmotic Pumps, model 2004, Durect Corporation) were implanted subcutaneously to administer Ang II (Sigma-Aldrich) at a dose of 1000 ng/kg/min for 28 days, and the abdominal aorta was harvested after 28 days of infusion. All experimental protocols were approved by the Institutional Animal Care and Use Committee of Tongji Medical College, Huazhong University of Science and Technology. All experiments also conformed to the guidelines of the Chinese Association of Laboratory Animals. Ang II (A9525, Sigma-Aldrich; 1000 ng/min/kg) or saline was administered to ApoE^−/−^ mice by osmotic mini-pump (model 2004, Alzet Osmotic Pumps) for up to 4 weeks. The mice were randomly assigned to 4 groups: Sham group (saline infusion + corn oil, 0.1 ml, orally administration daily after surgery, *n* = 8), Control group (saline infusion + ATRA-corn oil suspension, 0.1 ml, 3 days prior to surgery and orally administration daily after surgery, n = 8), AAA group (Ang II infusion + corn oil, 0.1 ml, orally administration daily after surgery, *n* = 12), and ATRA group (Ang II infusion + ATRA-corn oil suspension, 0.1 ml, 3 days prior to surgery and orally administration daily after surgery, n = 12). The ATRA-corn oil suspension was prepared at a dose of 5 mg/kg dissolved in 0.1 ml corn oil.

### ATRA treatment

ATRA is a drug that is typically used to treat acute promyelocytic leukemia. In the present study, All-trans-retinoic acid (ATRA, R2625, Sigma-Aldrich) was prepared from an ATRA-corn oil suspension immediately before use and protected from light. ATRA was administered to the Control group and the ATRA groups orally each day at a dose of 5 mg/kg (*n* = 8, 12) for 3 days prior to surgery. The mice continued to receive oral ATRA treatment for 4 weeks after surgery and were then sacrificed. The Sham group and the AAA groups were treated with the same protocol, except that an equivalent volume of corn oil was used in place of the ATRA-corn oil suspension.

### Blood pressure measurements

Blood pressure was measured 3 days before surgery (− 3 days), the day of surgery (day 0), and 3, 7, 14, and 28 days after surgery. All mice were awake, restrained, and maintained at a constant body temperature (36 °C) during the measurement of systolic blood pressure (SBP) by the noninvasive tail-cuff method (Softron BP-2010A).

### Aortic diameter measurement and aneurysms diagnosis

Prior to harvesting aortic samples, a micrometer was utilized to measure the maximum aortic diameter as previously described [18]. The increase in the suprarenal region of the abdominal aortic diameter was analyzed by digital photographs, and the micrometer was normalized to a ruler prior to each measurement. For diagnosing aneurysm formation, an aneurysm was defined as a ≥ 50% increase in the diameter of the suprarenal aorta compared with that in saline-infused mice or the occurrence of aortic dissection (AD).

### Histomorphometric study and immunohistochemistry

The details are described in the supplementary material.

### Western blot analysis

The details are described in the supplementary material.

### Statistical analysis

The data are expressed as the mean ± standard deviation, and groups were compared using the Mann-Whitney U test for nonparametric data or Student’s t-test for parametric data. The incidence of AAA was analyzed by the χ^2^ test. A commercially available statistical software package, SPSS 2.0, was used. For this study, *P* < 0.05 was defined as significant.

## Results

### Angiotensin II induces AAAs in ApoE^−/−^ mice, and treatment with ATRA reduces AAA incidence and mortality rate

**Fig. 1:**
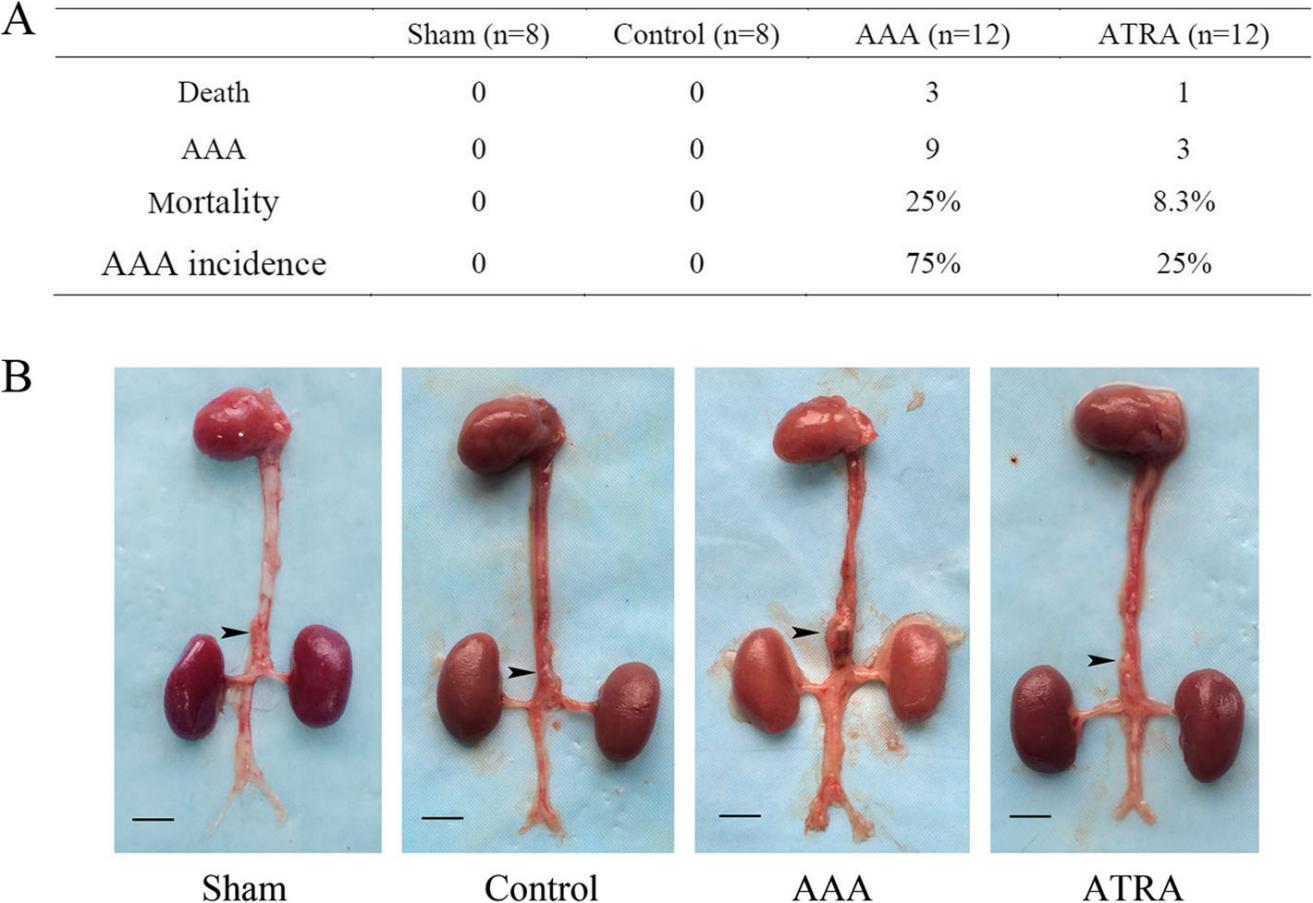
Mouse mortality and incidence of abdominal aortic aneurysms, and the heart and aorta gross appearance. **a**: ATRA reduces Ang II-induced AAA incidence and mortality in ApoE^−/−^ mice. Mortality = Number of deaths/Number of mice in each group. AAA incidence = Number of AAAs/Number of mice in each group. If a mouse died within 28 days, an autopsy was conducted to confirm massive hemorrhage caused by a ruptured abdominal aortic aneurysm. Mice were also diagnosed with AAA. **b**. Repressentative pictures of suprarenal abdominal aortic morphology identified in different groups. (as indicated by the black arrowheads). Scale bar is 5 mm

**Fig. 2:**
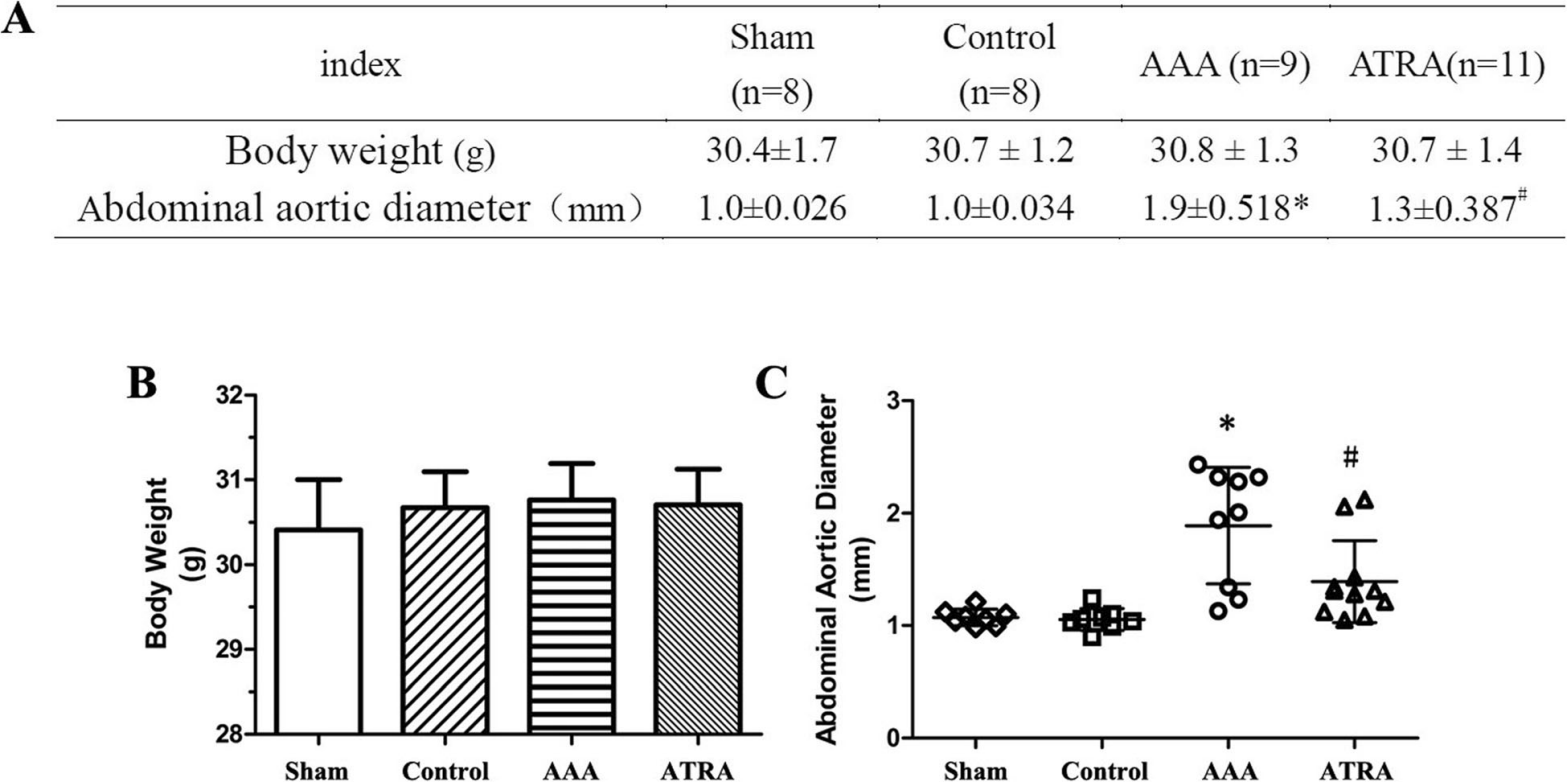
ATRA reduces the maximum diameter of the abdominal aorta but not body weight. **a** Body weight and abdominal aortic diameter. No statistically significant difference in body weight was noted. **b** There were no significant differences in body weight between the groups. **c**: Abdominal aortic diameter. Ang II infusion increased the aortic diameter in the AAA group. Compared with the AAA group, the aortic diameter was conspicuously decreased in the ATRA group. (AAA group, *n* = 9; ATRA group, *n* = 11). Data are the mean ± SD. ^*^P < 0.05 vs. Sham and Control group, ^#^*P* < 0.05 vs. AAA group

Three mice died from aortic dissection (AD) in the AAA group (3 of 12, 25%) one died from AD in the ATRA group (1 of 12, 8.3%), and no mice died in the Sham or Control groups during the 28-day experiment. Mice that died before day 28 were autopsied to confirm the occurrence of massive hemorrhage caused by a ruptured AAA. The model was therefore considered a success. The incidence of AAA in the AAA group was approximately 75% (9 of 12), which was significantly higher than that in the ATRA group (3 of 12, 25%, *P* < 0.05, Fig. 1a). During the experiment, there was no obvious difference in body weight among the Sham, Control, AAA, and ATRA groups (Fig. 2b). The AAA group exhibited balloon-like dilation in the abdominal aorta, whereas the Sham and Control groups did not exhibit any signs of AAA. AAAs were mainly observed in the suprarenal vessel of the abdominal aorta. We observed an amelioration of aneurysms in the abdominal aorta after ATRA administration (Fig. 1b). The abdominal aorta diameter data are presented in Fig. 2a. The size of the suprarenal abdominal aorta was measured with Image Pro Plus software, and it was significantly increased in the AAA group (*P* < 0.05, 1.9 ± 0.518 mm) compared to those in the ATRA (1.3 ± 0.387 mm), Sham (1.0 ± 0.026 mm) and Control groups (1.0 ± 0.034 mm), which had similar abdominal aortic diameters (Fig. 2c).

### Abdominal aortic pathology and immunohistochemistry

**Fig. 3:**
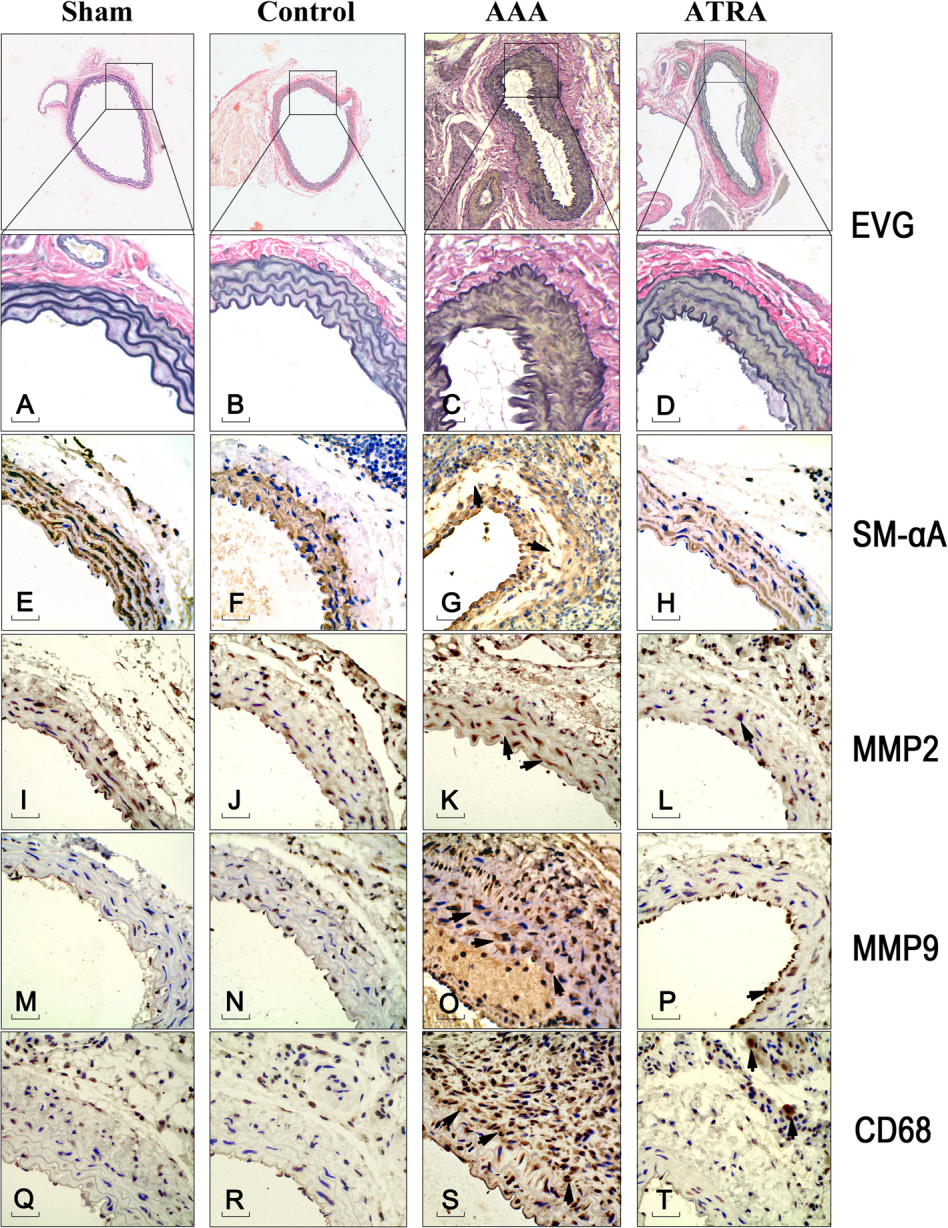
ATRA preserves the structure of the abdominal aortic wall and reduces MMP2, MMP9, and CD68 expression in abdominal aortic cross-sectional slices. **a-d**: EVG staining of the four groups. **e-t**: SM-αA, MMP2, MMP9, and CD68 immunohistochemical staining of different groups; the photographs show the immunohistochemical changes in the abdominal aorta (indicated by the black arrows). (A-X, × 400): Scale bar is 50 μm

Elastic fiber degradation and fracture and irregularly arranged cells were observed in the AAA group, as determined by Verhoeff-Van Gieson (EVG) staining (Fig. 3). These phenomena were not observed in the ATRA group. Serial sections were stained with antibodies against MMP2, MMP9, or smooth muscle-α actin (SM-αA) (positive staining for VSMCs) and CD68. In the AAA group, SM-αA staining revealed a partial defect in the medial vessel, which also displayed MMP2 and MMP9 expression and monocyte-macrophage infiltration. In the ATRA group, cells were arranged in an orderly manner, and elastic fiber integrity was preserved. ATRA protected smooth muscle cells (SMCs) and reduced MMP2, MMP9, and CD68 expression in the medial vessel. In the Sham and Control groups, the medial elastin fibers and SMCs were normal. The vascular wall showed faint staining for MMP2, MMP9, and CD68. These results indicate that ATRA regulates VSMC secretion of MMP2 and MMP9 and prevents monocyte-macrophage infiltration in the adventitia.

### Western blot analysis of RARα, MMP2, MMP9 and AT1 protein expression in the abdominal aorta

**Fig. 4:**
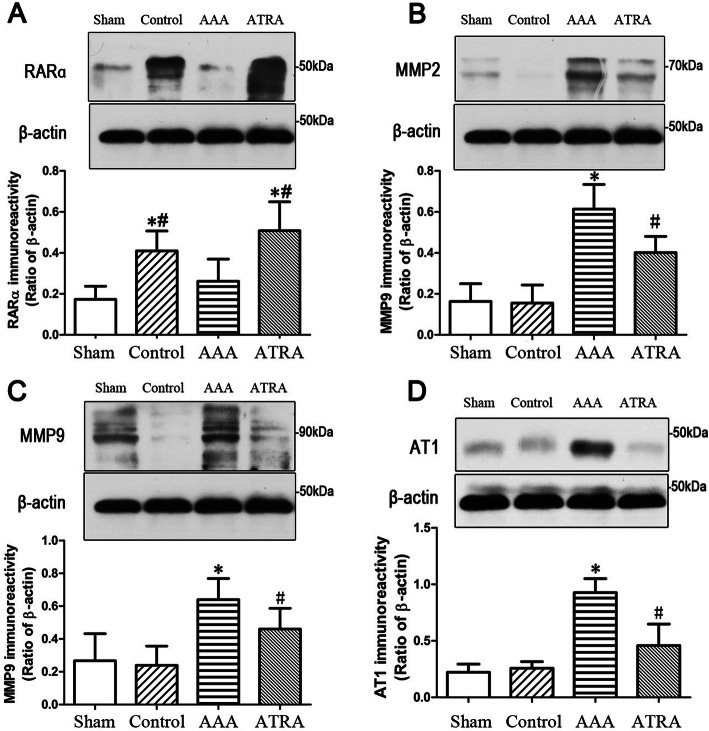
ATRA attenuates MMP2, MMP9, and AT1 protein expression in the abdominal aorta. **a**: ATRA induces RARα protein expression in the Control and ATRA groups. **b**, **c**, **d**: MMP2, MMP9, and AT1 expression was increased in the AAA group and significantly reduced in the ATRA group. Data are shown as the mean ± SD. ^*^P < 0.05 vs. Sham and Control group, ^#^*P* < 0.05 vs. AAA group, *n* = 5 per group

ATRA upregulated RARα protein expression and decreased MMP2, MMP9, and AT1 protein expression in the abdominal aorta in Ang II-treated ApoE^−/−^ mice. Our results clearly showed that RARα expression was upregulated by ATRA in the Control and ATRA groups (Fig. 4a). However, differences in expression of MMP2, MMP9, and AT1 in the AAA and ATRA groups were highly significant (Fig. 4b, c, d). These results indicate that ATRA induced the overexpression of RARα and reduced the expression of MMP2, MMP9, and AT1 in the AAA group, which suggests that ATRA maintains vascular integrity through RARα and plays a negative regulatory role in AAA. However, no difference was observed in the abdominal aortae in the Sham or Control groups.

### Blood pressure measurements

**Fig. 5:**
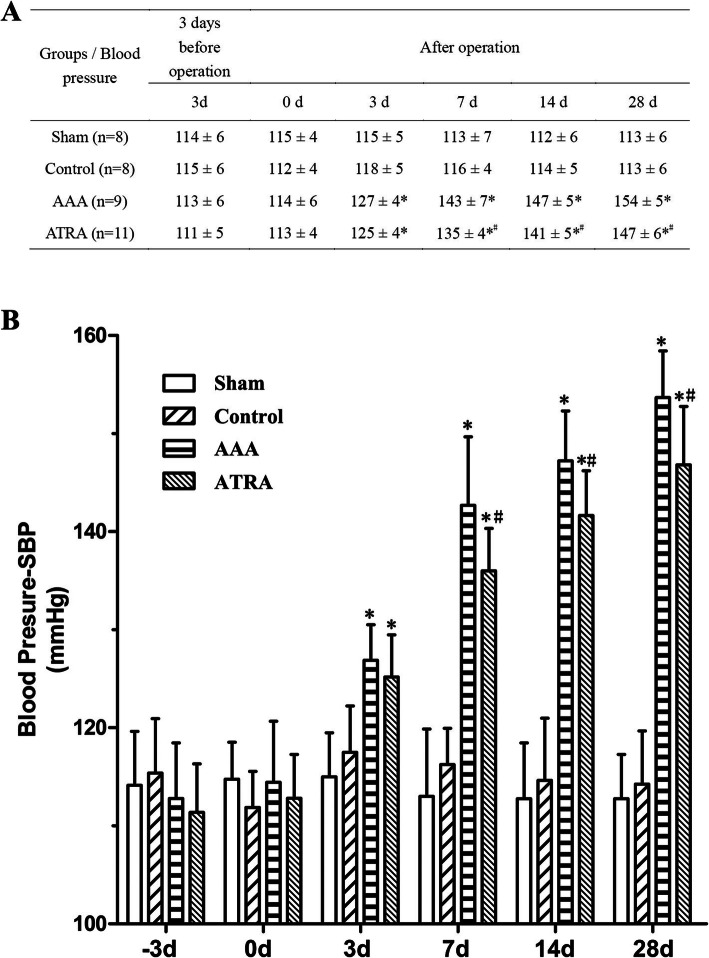
ATRA appears to lower systolic blood pressure at 7, 14, and 28 days after operation. **a**: Results of Systolic blood pressure. **b**: There was no significant difference between the systolic blood pressure of the Sham and Control groups. A obvious decrease in systolic blood pressure (SBP) was observed in the ATRA group compared to the AAA group at 7, 14, 28 days after operation. Data are shown as the mean ± SD.^*^P < 0.05 vs. Sham (*n* = 8) and Control groups (n = 8), ^#^*P* < 0.05 ATRA group (n = 11) vs. AAA group (n = 9)

A marked decrease in systolic blood pressure (SBP) was observed in the ATRA group compared to that in the AAA group (*P* < 0.05) (Fig. 5a and b). There was no difference in SBP between the AAA and ATRA groups at − 3 days, 0 days, or 3 days after ATRA treatment. However, the SBP of the ATRA group was effectively reduced at 7 days, 14 days, and 28 days after ATRA treatment (*P* < 0.05). No statistically significant change was observed in SBP between the Sham and Control groups. No statistically significant difference in body weight was observed among the 4 groups at 28 days after treatment, indicating that ATRA treatment reduced blood pressure in Ang II-treated ApoE^−/−^ mice but had no effect on body weight.

## Discussion

Abdominal aortic aneurysm (AAA) is a local manifestation of a systemic vascular disease. Although the disease processes are different, AAA has similar risk factors as atherosclerosis, including age, hypertension, male sex, smoking and hypercholesterolemia. Surgical intervention may be the only effective strategy for AAA patients, and no effective drug treatments exist. The main effects of drug prevention included reducing blood pressure, cholesterol, and inflammation. Therefore, effectively inhibiting the development of the small AAAs and preventing AAA rupture are the main goals of drug treatment.

In the present study, we examined the effect of ATRA administration on the maximum transverse diameter of the abdominal aorta and the incidence of AAA. We observed a lower incidence of AAA in the ATRA group than in the AAA group (3 of 12 (25%) vs. 9 of 12 (75%), χ^2^ = 6.0001*, P* = 0.0143). We explored the potential mechanism by which ATRA reduces the development of AAAs through histopathological analysis, immunoblotting experiments, and blood pressure evaluation.

In the AAA group, elastic fiber disruption and decomposition and the disordered arrangement of smooth muscle cells were observed by EVG staining. In contrast, the ATRA group had vascular wall integrity, which demonstrates that the ATRA has a protective effect in the vascular wall. The observed elastic fiber disruption was most likely caused by the secretion of MMP2 and MMP9, which were detected by immunohistochemical staining. MMP2 and MMP9 are closely associated with AAA because AAA formation is reduced when the genes encoding these proteins are knocked out in mice [13]. These data suggest that MMP2 and MMP9 play an important role in the development of AAAs. Similarly, tissue inhibitors of MMPs (TIMPs) are known to have an antagonistic effect on MMPs [19]; overexpression of TIMPs inhibit MMP function, while knockout of the TIMP-1 gene increases the occurrence of AAAs [20]. Furthermore, the infiltration of vessel walls by large numbers of monocyte-macrophages also induces the secretion of MMPs [21], resulting in the destruction of elastic fibers and thus promoting AAAs. In our study, ATRA treatment reduced monocyte-macrophage infiltration of the vascular adventitia. The inhibition of MMP2 and MMP9 activity by ATRA reduced elastic fiber destruction, and the elastic fiber continuity in the ATRA group was similar to that in the Sham and Control groups.

We confirmed the above results by Western blotting, which demonstrated that ATRA administration increased RARα expression in the abdominal aorta. Furthermore, the protein expression of MMP2, MMP9 and AT1 was increased after 28 days of infusion, and treatment with ATRA downregulated the protein expression of MMP2, MMP9 and AT1. These results confirmed that increased AT1 expression is correlated with AAAs, which was similar to the results of the Habashi group showing that AT1 antagonist treatment reduced the occurrence of AAAs [22]. Furthermore, Haxsen and colleagues found that ATRA downregulated AT1 mRNA levels, which indicated that retinoic acid may interfere with Ang II-mediated VSMC proliferation [23]. Moreover, our results revealed that increased expression of the RARα protein was negatively correlated with MMP2, MMP9, and AT1 expression. ATRA alleviated the degradation of elastic fibers caused by MMP2 and MMP9, indicating an important role for ATRA in maintaining vascular integrity. A previous study reported that β-carotene, a retinoic acid precursor, can reduce macrophage infiltration and inhibit the expression of MMP2, MMP9, and MMP12 through RAR binding to peroxisome proliferator-activated receptors (PPARs), thereby slowing AAA formation [24].

The potential mechanism by which ATRA reduces AT1 expression may occur through the APJ (a putative receptor protein related to AT1) pathway [25]; this reduction in AT1 expression reduces the impact of Ang II-induced inflammatory infiltration and hypertension on blood vessel walls. Indeed, treating hypertension is beneficial for preventing AAAs. In our study, Ang II infusion increased blood pressure to a certain degree compared with saline infusion, 3 mice died in the AAA group, and 1 mouse died in the ATRA group due to the AD. In our opinion, the Ang II-induced hypertension in the mice may be responsible for the occurrence of AD and death. However, Cassis [26] found that infusions of Ang II and norepinephrine promoted similar increases in blood pressure but had different effects on AAA and indicated that attenuation of Ang II-induced increases in blood pressure had no effect on AAA incidence. Further research is needed to clarify this hypothesis. In addition, Zhong and his teammates found that ATRA can reduce the expression of AT1 and increase angiotensin-converting enzyme 2 (ACE2) expression [27]; both of these changes in protein expression reduce blood pressure in spontaneously hypertensive rats (SHRs). Blood pressure is also regulated by the RAR/RXR-mediated endothelial-dependent NO-cGMP pathway [28]. In the current study, Ang II upregulated the expression of AT1, thus increasing blood pressure. In the AAA and ATRA groups, blood pressure began to increase 3 and 7 days after surgery. The average blood pressure in the AAA and ATRA groups was 140 mmHg or higher, which was significantly higher than the SBP in the Sham and Control groups. The SBP in the ATRA group, which was approximately 135 mmHg, did not increase as much as that of the AAA group, suggesting that ATRA can reduce blood pressure by abrogating the increase in AT1 in Ang II-stimulated VSMCs. After 7, 14, and 28 days, the ATRA group displayed a 5–10 mmHg decrease in mean SBP compared to the AAA group. Thus, ATRA administration might reduce the occurrence of AAA by downregulating the expression of not only MMP2 and MMP9 but also AT1, thereby lowering blood pressure and reducing the load on the vessel wall.

There are many ATRA receptors, including RARα, β, and γ and RXRα, β, and γ. In this sutdy, only RARα expression was analyzed to determine the effects of ATRA on vessels. Future studies should examine the effects of RARα agonists or antagonists, as well as the role of RARβ and γ and RXRα, β, and γ, in AAAs. In conclusion, this demonstrates that ATRA administration may attenuate AAA formation and progression, suggesting a new therapeutic option for treating or preventing AAAs.

## Conclusions

In conclusion, ATRA attenuates the progression of Ang II-induced AAAs, possibly by downregulating MMP2, MMP9, and AT-1 expression.

## Supplementary information

**Additional file 1.**

## Data Availability

The datasets used and/or analyzed during the current study are available from the corresponding author upon reasonable request.

## Abbreviations

Ang II: Angiotensin II
AAA: Abdominal aortic aneurysm
ApoE^-/-^: Apolipoprotein E knock-out
ATRA: All-trans retinoic acid
H&E: Hematoxylin-eosin
EVG: Verhoeff-Van Gieson
AT1: Angiotensin II receptor type 1
MMP-2: Matrix metalloproteinase 2
MMP-9: Matrix metalloproteinase 9
VSMCs: vascular smooth muscle cells
RARs: Retinoic acid receptors
