# Supplementary material for: All-trans retinoic acid attenuates the progression of Ang II-induced abdominal aortic aneurysms in ApoE−/−mice
Source: J Cardiothorac Surg. 2020 Jul 2;15:160. doi: 10.1186/s13019-020-01208-w (PMC7331218; doi:10.1186/s13019-020-01208-w)
Supplement: Supplementary file 1 — Additional file 1. [file 13019_2020_1208_MOESM1_ESM.docx]

**Histomorphometric study and immunohistochemistry**

Twenty-eight days after subcutaneous osmotic pumps were implanted, the mice were weighed and anesthetized with by injection of 2% pentobarbital sodium into the abdominal cavity (30 mg/kg), and the thoraco-abdominal aortae and hearts were exposed. Using an injection syringe inserted into the apex of the heart, tissues were initially flushed with phosphate-buffered saline (PBS) and then fixed by continuous perfusion of the vessels with 4% paraformaldehyde at physiological pressure, approximately 100 mmHg. The tissues were then embedded transversely in paraffin and sectioned at a thickness of 4 µm. Cross-sections were stained by elastica-van Gieson elastin staining. For morphological analyses, images of EVG-stained cross-sections stained were recorded with a camera connected to a light microscope at 100× and 400× magnifications. The medial area was defined as the area between the external and internal elastic plate. Immunohistochemical staining was performed on serial paraffin-embedded sections with primary antibodies against MMP2 (sc-10736, 1:100 dilution, Santa Cruz Biotechnology), MMP9 (sc-10737, 1:100 dilution, Santa Cruz), Smooth muscle-α actin (SM-αA, BM0002, 1:100 dilution, Boster), and CD68 (AB125047, 1:100 dilution, Abcam). The abdominal aortic diameter was measured by computer-assisted morphometry analysis software with Image Pro Plus 6.0. For each of the analyses described above, a section from each mouse aorta was randomly selected.

**Western blot analysis**

Protein lysates were prepared from each mouse abdominal aortic tissue sample, which was collected 28 days after surgery. The samples were homogenized in protein lysis buffer on ice (Beyotime, Nantong, China) in 1 mM phenylmethylsulfonyl ﬂuoride (PMSF) buffer supplemented with a protease inhibitor cocktail and phosphatase inhibitor cocktail for 30 minutes, followed by centrifugation at 17,000 rpm for 15 minutes at 4°C. The protein concentration of each sample was determined using a bicinchoninic acid assay (Sigma, St Louis, MO). Samples (30 μg of protein) were electrophoresed on a 10% sodium dodecyl sulfate-polyacrylamide gel at 80 V and transferred to a polyvinylidene diﬂuoride (PVDF) membrane at 300 mA. The membranes were blocked for 2 hours at room temperature with 5% nonfat milk in Tris-buffered saline containing 0.2% Tween 20. The membranes were incubated overnight at 4°C with the following primary antibodies: β-actin (1:2000 dilution; SC-1616R, Santa Cruz Biotechnology), RARα (1:800 dilution; SC551, Santa Cruz Biotechnology), MMP2 (1:2000 dilution; SC373914, Santa Cruz Biotechnology), MMP9 (1:2000 dilution; SC10737, Santa Cruz Biotechnology), and AT1 (1:2000 dilution; SC1173, Santa Cruz Biotechnology). The membranes were washed in Tris-buffered saline and incubated for 2 hours at room temperature with horseradish peroxidase-conjugated (HRP)-conjugated sheep anti-mouse secondary antibodies (1:2000 dilution; KPL, Kirkegaard & Perry Laboratories, Inc.). The expression signals were visualized by electrochemiluminescence (ECL) staining, and the bands were scanned and quantified using AlphaEaseFC software. Protein expression was normalized to β-actin expression.
